# Avoiding Compartment Syndrome, Vascular Injury, and Neurologic Deficit in Tibial Osteotomy: An Observational Study of 108 Limbs

**DOI:** 10.5435/JAAOSGlobal-D-23-00075

**Published:** 2023-11-16

**Authors:** Jason Shih Hoellwarth, Adam Geffner, Austin T. Fragomen, Taylor J. Reif, S. Robert Rozbruch

**Affiliations:** From the Limb Lengthening and Complex Reconstruction Service (LLCRS). Hospital for Special Surgery. New York, NY.

## Abstract

**Introduction:**

Tibial deformities are common, but substantial concern may be associated with corrective osteotomy regarding major complications reported in classic literature. Such studies chiefly focused on high tibial osteotomy, with relatively little investigation of other areas and types of deformity. The primary aim of this study was to identify the rate of compartment syndrome, vascular injury, nerve injury, and other major complications after elective tibial osteotomy.

**Methods:**

One hundred eight tibia osteotomies performed during 2019 to 2021 were evaluated, representing all tibia osteotomies except situations of existing infection. A retrospective chart review was performed to identify patient demographics, surgical indications, anatomic location of osteotomy, fixation used, and complications prompting additional surgery.

**Results:**

The most common osteotomy locations were high tibial osteotomy (35/108 = 32%, 32/35 = 91% medial opening, and 3/35 = 9% medial closing), proximal metaphysis (30/108 = 28%), and diaphysis (32/108 = 30%). The most common fixation was plate and screw (38/108 = 35%) or dynamic frame (36/108 = 33%). Tranexamic acid was administered to 107/108 = 99% of patients and aspirin chemoprophylaxis was used for 83/108 = 86%. A total of 33/34= 97% of anterior compartment prophylactic fasciotomies were performed for diaphyseal or proximal metaphysis osteotomies. No events of compartment syndrome, vascular injury, nerve injury, or pulmonary embolism occurred. One patient required débridement to address infection. Additional surgery for delayed/nonunion occurred for nine segments (8%). Additional surgery for other reasons were performed for 10 segments (9%), none resulting in reduced limb function.

**Conclusion:**

Tibial osteotomy can be safely performed for a variety of indications in a diverse range of patients, without a notable risk of the most feared complications of compartment syndrome, vascular injury, and neurologic deficit. Prophylactic fasciotomy and reducing postoperative bleeding using tranexamic acid, along with location-specific safe surgical techniques, may help prevent major complications and thereby facilitate optimized deformity care.

Tibial osteotomy can be indicated for the correction of many lower extremity deformities, prompting the use of various fixation hardware (Figure 1). The high tibial osteotomy (HTO) to address coronal plane deformity about the knee is a common example. Osteotomy at other locations may be indicated to address sagittal plane, rotational, or length deformities. Early literature expressed concern regarding the rate of major complications when performing tibial osteotomy: compartment syndrome, vascular injury, persistent neurologic injury,^1,3^ and even death.^4^ Although improvements of surgical technique and technology have improved safety,^5,6^ the rate of such complications remains non-negligible.^7^ Because of lingering concerns regarding major complications reported in early investigations of tibia osteotomy,^8^ some surgeons may be hesitant to electively correct tibial deformities or may consider performing the osteotomy at a different anatomic site than the otherwise preferred bony location. Perhaps because of this apprehension, with the exception of HTO,^9^ limited contemporary literature exists describing complications of and techniques which maximize safety for tibial osteotomies.

**Figure 1:**
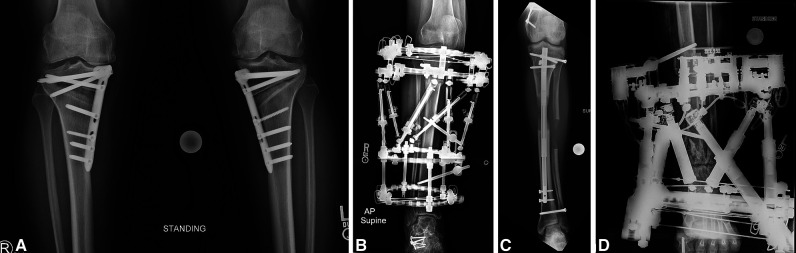
Radiographs showing the examples of osteotomies and fixation included in this study. **A,** A 52-year-old woman with staged bilateral high tibia osteotomies, fixated with plate and screws, to address tibia-based genu varum; she did not have any fasciotomy. **B,** A 65-year-old woman with right proximal metaphyseal tibia osteotomy and mid-diaphyseal fibula osteotomy, fixated with a dynamic hexapod external fixator, to address torsion; she had an anterior compartment fasciotomy. **C,** A 21-year-old man with left diaphyseal tibia and fibula osteotomy, fixated with dynamic nail, to address short stature related to hypophosphatemic rickets; he had anterior and lateral compartment fasciotomies. **D,** A 41-year-old woman with left supramalleolar tibia and fibula osteotomy, fixated with a dynamic hexapod external fixator, to address a multiplanar deformity; she did not have any fasciotomy. The radiographically dense struts and wires are part of a robotically self-controlled programmable adjustment system.

This study was performed to help address that knowledge gap. The primary aim was to evaluate the major complications that occurred after tibial osteotomy performed at various anatomic locations for a diverse variety of indications.

## Methods

Afte institutional ethics approval, a retrospective chart review was performed of our practice's surgical registry. Inclusion criteria were tibia osteotomy (any location, any indication) with follow-up through the conclusion of care, performed during the years of 2019 to 2021. Exclusion criteria were tibia osteotomy performed in the setting of existing infection (five limb segments). This yielded 108 limb segments (87 patients).

A chart review identified demographics, indication for surgery, tibial osteotomy location, fixation hardware used, whether the fibula was osteotomized, whether tranexamic acid (TXA) was administered, whether (and which) prophylactic compartment fasciotomy was performed, and the venous thromboembolism chemoprophylaxis regimen.

Outcomes evaluated were major complications: compartment syndrome, vascular injury, nerve injury, infection, delayed/nonunion, broken hardware, other issues prompting surgical intervention, deep vein thrombosis (DVT), and pulmonary embolism (PE).

The radiology picture archiving and communication software used for planning the osteotomies was Sectra picture archiving and communication software (Sectra). Hexapod frame adjustment software used was that provided by the specific device manufacturer, depending on which hexapod product was used for each individual case. Descriptive statistics were calculated using Google Sheets (Google LLC). Comparative statistical analysis was not performed.

A full description of techniques used for the osteotomies summarized herein is beyond the scope of this manuscript. However, the following principles and considerations were essential and universally applicable. HTOs were approached through a 12-cm longitudinal incision medially, with the cut trajectory from the proximal metaphyseal flare toward the lateral plateau corner guided by a 2-mm guidewire placed centrally. A pneumatic powered oscillating saw with a 40-mm long blade made the initial cuts through the central and posterior tibial cortex; the anterior trajectory preserved the tibial tubercle with the distal tibia by changing the saw trajectory to obliquely cut through the plateau anterior to the joint surface but posterior to the tubercle. Metal retractors protected the posterior soft tissues and also the patellar tendon. Long, thin osteotomes without a continuously widening taper were used to extend the osteotomy toward the lateral subplateau but preserving the lateral hinge, and laminar spreaders were used to achieve the opening gap as needed, which was then filled with allograft wedge and bone void filler paste and stabilized with a locking plate. Fibula osteotomy was never performed for HTOs, and neither were peroneal nerve releases. Complete osteotomies (those without a hinge) were performed by first drilling multiple paths along the trajectory of the osteotomy with a solid core drill bit appropriate for the size of the bone, generally 4 to 5 mm diameter. These osteotomies were completed with manual osteotomes. Manual string-style (Gigli) saws or powered saws were not used, to minimize heat generated and potential associated thermal injury to the bone. Retractors generally were not used for manual osteotome osteotomies because these incisions were generally small. Local soft-tissue protection was achieved by ensuring the osteotome was firmly against bone before impacting, carefully listening to sound changes, gently performing wrench-assisted osteotome rotation as the osteotomy neared completion. In all surgeries, the entire leg was supported above and below the osteotomy, generally with at least two bumps made from sterile towels wrapped in sterile adhesive drape or elastic bandage. These bumps were critically important to prevent the leg from dropping or otherwise rapidly changing gross position upon completion of the osteotomy, which could lead to acute stretch or even laceration against a cut bone surface. Fasciotomies were performed using the full length of a compartment using a fasciotome featuring an “elbow” along its length to slide beneath the skin.

## Results

Table 1 presents the demographics and surgical variables. The average age was 38.0 ± 15.6 (range, 5.7 to 72.8) years. Men represented 57/108 = 53%. Notable aspects include the near-complete rate of TXA administration (107/108 = 99%) and the very high rate of aspirin for DVT chemoprophylaxis (93/108 = 86%). The three most common anatomic locations for osteotomy were the high tibia (35/108 = 32%, 32/35 = 91% medial opening, 3/35 = 9% medial closing), proximal metaphysis (30/108 = 28%), and diaphysis (32/108 = 30%). Fixation was mostly provided with plate and screw (38/108 = 35%) or dynamic external frame (36/108 = 33%). Regarding prophylactic fasciotomy, 33/34 = 97% of anterior releases were performed for patients with a single diaphyseal (26/32 = 81%) or proximal metaphysis (7/30 = 23%) osteotomy; the other fasciotomy was performed for a patient who had multiple osteotomy locations (1/3 = 33%). Specifically, 0/35 = 0% of HTOs had fasciotomy. Myriad fixation options were used for patients who had anterior fasciotomy: 3/34 = 9% had dynamic frames, 20/34 = 59% had static intramedullary nails, 10/34 = 29% had dynamic nails, and 1/34 = 3% had plate and also nail fixation. Lateral compartment release was performed for 16/65 = 25% of fibula osteotomies (none were performed without an associated fibula osteotomy). The tibial osteotomy was performed in the diaphysis for 15/16 = 94% of lateral fasciotomies, with the remaining one associated with a proximal metaphysis osteotomy.

**Table 1:** Patient Demographics and Surgical Variables

Variable	Data: no. (% of 108) (fasciotomies)
Anterior fasciotomy	34 (32%)
Lateral fasciotomy	16 (15%)
Fibula osteotomy	65 (60%)
Intraoperative TXA	107 (99%)
Chemoprophylaxis	
None	3 (3%)
Aspirin	93 (86%)
Rivaroxaban	8 (7%)
Multiagent	4 (4%)
Osteotomy location	
High tibia	*35 (32%) (0)*
Proximal metaphysis	*30 (28%) (7, 23%)*
Diaphyseal	*32 (30%) (26, 81%)*
Distal metaphysis	*2 (2%) (0)*
Supramalleolar	*6 (6%) (0)*
More than one location	*3 (3%) (1, 33%)*
Fixation	
Plate and screw	38 (35%)
Dynamic frame	36 (33%)
Static frame	20 (19%)
Dynamic nail	12 (11%)
Plate and screw plus dynamic nail	2 (2%)
Indication	
High tibia varus/valgus	*30 (28%) (0)*
Supramalleolar varus/valgus	*1 (1%) (0)*
Other varus/valgus	*15 (14%) (4, 27%)*
Biplanar	*22 (20%) (11, 50%)*
Multiplanar	*13 (12%) (2, 15%)*
Lengthening	*12 (11%) (5, 42%)*
Torsion	*8 (7%) (7, 88%)*
Malunion	*5 (5%) (4, 80%)*
Procurvatum/recurvatum	*2 (2%) (1, 50%)*

TXA = tranexamic acid.

The primary outcome investigated was major complications, which are itemized in Table 2. None of the 108 patients had any of the most concerning complications: compartment syndrome, vascular injury, or nerve injury. Two patients developed a DVT (2%), but none experienced a PE. One patient (1%) required surgery to address infection: a 27-year old woman with a left multiplanar deformity treated with a hexapod developed infection persistent after frame removal, which resolved after a soft-tissue débridement.

**Table 2:** Major Complications

Overall Complications	Occurrences
Compartment syndrome	0
Vascular injury	0
Nerve injury	0
Infection surgery	1 (1%)
Delayed/nonunion	8 (7%)
Broken hardware	0
Other additional surgery	11 (10%)
DVT	2 (2%)
PE	0

DVT = deep vein thrombosis, PE = pulmonary embolism.

Table 3 outlines the interventions for delayed/nonunion, and Table 4 outlines other situations of unplanned surgery. Nine segments (8%) in eight patients had intervention for delayed or nonunion. Five of the segments required relatively minor interventions to stimulate the bone in the osteotomy site, such as percutaneous wire drilling of the regenerate, bone marrow aspirate injection, placement of bone morphogenetic, or nail dynamization. Ten segments (9%) in nine patients had additional surgery for other reasons (Table 4). Most of these were relatively minor (hematoma evacuation, repeat osteotomy, distal tibia-fibula stabilizing screw, gastroc-soleus recession for equinus during lengthening, repeat osteotomy for preconsolidation, or supplemental frame fixation). More substantial surgeries were one revision fixation for malalignment and two patients who fractured between hardware or after hardware removal, prompting osteosynthesis nailing.

**Table 3:** Summary of Delayed/Nonunion

Age Sex	Side, Indication, Location, Fixation	Management
30 women	Right, multiplanar deformity, proximal metaphysis, static nail	Percutaneous wire drilling
65 women	Right, torsion and short, proximal metaphysis, hexapod frame	Supplemental ring fixation
22 men	Bilateral, stature lengthening, diaphysis, MILN	Bone marrow aspirate injection
27 men	Right, multiplanar deformity, multiple osteotomy locations, plate/screw	Bone marrow aspirate concentrate injection with bone morphogenic protein-2 sponge carrier
35 women	Right, torsion, diaphysis, static nail	Dynamization
56 women	Left, short, diaphysis, MILN	Bone marrow aspirate concentrate injection with bone morphogenic protein-2 sponge carrier, with subsequent exchange nailing
23 women	Left, biplanar deformity, proximal metaphysis, static nail	Percutaneous drilling, bone marrow aspirate concentrate injection, autograft from Gerdy tubercle
54 women	Left, biplanar deformity and short, proximal metaphysis, MILN	Conversion to hexapod frame to improve stability and achieve correction

MILN = motorized intramedullary lengthening nail.

**Table 4:** Summary of Additional Surgery for Other Reasons

Age Sex	Side, Indication, Location, Fixation	Complication	Management
34 men	Left, genu valgum, HTO, plate/screw	Hematoma	Evacuation
55 men	Right, genu valgum, HTO, plate/screw	Malalignment	Revision fixation
22 men	Bilateral, stature, diaphysis, MILN	Fibula migration	Distal tibia-fibula screw fixation
31 men	Left, leg length discrepancy, diaphysis, MILN	Fracture at osteotomy after hardware removal	Solid nailing
58 men	Right, coronal plane deformity, hexapod frame	Insufficient frame stiffness	Additional frame stabilization
61 women	Right, leg length discrepancy, diaphysis, MILN	Fracture between lengthening nail and fused ankle	Lengthening nail removal and hindfoot nailing
5 women	Right, leg length discrepancy, proximal metaphysis, MILN	Ankle equinus	Gastroc-soleus recession
51 women	Left, leg length discrepancy, diaphysis, MILN	Premature consolidation	Repeat osteotomy
54 women	Left, biplanar deformity and short, proximal metaphysis, MILN	Loss of deformity correction	Exchanged to hexapod

HTO = high tibial osteotomy, MILN = motorized intramedullary lengthening nail.

## Discussion

This study had the primary aim to evaluate the rate of major complications after elective tibial osteotomy. The most important finding is that osteotomies can be performed in many anatomic areas of the tibia, for a variety of indications, using myriad fixation techniques, while avoiding the most catastrophic major complications: compartment syndrome, vascular injury, nerve injury, and PE. No events of these occurred in our cohort of 108 limb segments. Interventions for less calamitous issues such as delayed or nonunion, peri-implant fracture, and other adverse events associated with deformity care did occur, but none led to permanent disability. In our opinion, this suggests that surgeons with training in and experience with safe tibial osteotomy techniques should feel empowered to correct tibial deformity at the appropriate anatomic location, without excessive concern that a serious complication is likely to result. Understanding the situations which increase risk, and mitigating the risk via techniques or technology, is critical to optimizing safety.

Other than death, compartment syndrome is likely the most concerning complication associated with tibial osteotomy. Compartment syndrome can occur from insufficient inflow of oxygen and nutrition or reduced outflow of deoxygenated blood and metabolites. Examples include acutely reducing compartment volume with deformity correction or increasing compartment pressure (bleeding). A fasciotomy expands the volume available for intracompartmental anatomy, reducing pressure. A limited incision approach may be insufficient in acute compartment syndrome^10^ but is often sufficient when performed for prophylactic situations,^11^ including tibial osteotomy.^5,8,12^ Prophylactic fasciotomy for lengthening was reported by 1978,^12^ and prophylactic fasciotomy has rarely caused detectable muscle injury.^13^ When not routinely performing compartment release, Krengel and Staheli^14^ reported a rate of 2/39 = 5% compartment syndrome for proximal tibial osteotomy versus 0/13 = 0% for distal tibial osteotomy. When routinely performing prophylactic anterior fasciotomy, Stotts and Stevens^11^ reported no compartment syndrome in 59 tibial rotational osteotomies for adolescents and young adults. Our group's main indication for anterior compartment fasciotomy is acute deformity correction, in any plane, except in HTO. In particular, we routinely perform prophylactic anterior fasciotomy when imparting acute correction in the meta-diaphyseal or diaphyseal tibia. Gradual-only correction such as with a lengthening nail or frame is not usually an indication for fasciotomy. We feel that a lateral compartment release is unnecessary without a fibula osteotomy and may be most useful for acute rotation. Complete longitudinal compartment releases can be performed through a 1-cm incision using a fasciotome featuring an “elbow” bend, advanced antegrade, and retrograde. Fasciotomy with scissors is more difficult and may injure adjacent structures.^15^

Preventing bleeding reduces intracompartmental pressure. Preoperative TXA does this effectively and safely with minimal recognized risk,^16^ specifically including tibial osteotomy.^9^ A second dose around four hours after the first dose provides additional benefit.^17^ Few contraindications other than medication allergy^18^ or perhaps a history of seizures^19^ have been recognized. We gave TXA to 107/108 = 99% of this study's patients; no TXA-related adverse events occurred. One patient did not receive TXA because of a concern for inducing a blood clot; in retrospect, this likely need not have been a contraindication. Although this study is not designed or intended to evaluate the specific impact of TXA alone on preventing compartment syndrome or other complications, we believe the apparent safety benefit likely substantially outweighs the risk. This is based on our data identifying no adverse events apparently attributable to TXA in our cohort and also the aforementioned literature explicitly identifying the benefits of TXA with very minimal risks. Additional considerations to limit bleeding include injectable bone void fillers tamponade the cut bone edges and drains to provide egress of extravasated blood.^8^

Besides compartment syndrome, a vascular injury, specifically arterial injury, is another serious complication. Voluminous literature describes popliteus arterial injury risk during HTO, including these two which explore the relevant anatomy both with and without fibular osteotomy.^20,21^ In the proximal fibula, the anterior tibial artery and peroneal artery are vulnerable to tibial and fibular osteotomies.^22^ Reports of arterial injuries beyond the proximal tibia are rare,^23^ particularly in the supramalleolar region.^24^ Percutaneous osteotomy technique featuring predrilling optimizes cosmesis and soft-tissue integrity but impairs visibility of neurovascular anatomy. Drilling with two hands,^25^ using a sharp and well-designed drill bit spinning at full speed,^26^ optimizes tactile feedback to avoid plunging and produces lower temperatures.^27^ Accessory incisions, to improve visibility of the bone, allow careful^28^ predrilling or achieve direct access to improve safety when a single approach is limiting.^29^ Similarly, when using a saw during an open approach, favorable positioning (knee flexion to move the popliteus artery posterior) is easy and recommended but unreliable on its own,^20^ and anatomic variation occurs.^30^ Especially for HTO, encircling the bone with retractors prevents adjacent soft-tissue laceration, not only from the saw blade tip but also from the oscillations,^31^ which can cause arterial pseudoaneurysm even if true laceration does not occur.^23^ Irrigation during drilling and sawing reduces temperature^32^ but can obscure visibility so may be more convenient in spurts between active power utilization.

There are other arterial safety considerations. Acute shortening can kink arteries,^33^ at or before 4 cm^34^; in such situations, frequent pulse checks are important. Acute angular or rotational correction can also constrict arteries, which can present as a secondary neurologic dysfunction.^3^ Vessel injury from sudden position change on osteotomy completion^35^ can be prevented by supporting the extremity above and below. External fixation can achieve gradual correction, either during surgery or subsequently, preventing vessel kinking. The fixator can remain for the duration of healing or can be exchanged for a plate or a nail.^36^

Nerve injuries are a third major potential complication. The bulk of nerve injuries are also reported regarding HTO, as an acute varus correction of valgus deformity stretches the common peroneal nerve.^37^ Any stretch either acutely or during the course of correction can induce a nerve palsy.^38^ Nerve protection principles are similar to arterial principles, with the following fundamentals optimizing safety. Peroneal nerve release about the fibular head^39^ can be performed prophylactically or therapeutically. Our group's main indication for peroneal nerve decompression is acute correction of valgus and/or external torsion in the diaphysis or metadiaphysis. The tibial nerve is most often stressed distally after correction of ankle varus, equinus and/or procurvatum, and in lengthening situations. Prophylactic or therapeutic release of the entire tarsal tunnel^40^ is beneficial, especially if acute bony correction induces equinus, prompting tendoachilles lengthening or gastrocnemius-soleus complex recession. Our group's main indication for tibial nerve decompression via a tarsal tunnel release is the acute correction of varus and/or internal torsion in the distal metaphysis or metadiaphysis. It deserves specifically reiterating that an apparent neurologic deficit may actually be because of a primary arterial compromise.^3^

Although it is hoped that this study lends support to the safety of tibial deformity correction when best technique and technology practices are used, there are important limitations to consider. The primary may be the generalizability of the outcomes. The surgeons who performed these procedures all focus on deformity correction and have had specific fellowship training in the techniques and principles described; not all surgeons have this familiarity. Another potential limitation is the unequal number of procedures performed at any given osteotomy level or with any given fixation option, limiting equal data interpretation. In addition, as a retrospective study, it is possible that subclinical changes in sensation or motor function may not have been detected. A major strength of the study is the variety of deformity indication, osteotomy location, and fixation type. No events of compartment syndrome, arterial injury, or nerve dysfunction occurred despite the broad range of patient ages, indications, and fixation options supports the utility of the principles and techniques espoused.

## Conclusions

Tibial osteotomy can be safely performed throughout the tibia to manage a variety of indications without causing major complications such compartment syndrome, vascular injury, and neurologic deficit. Optimizing safety with specific surgical techniques, reducing bleeding, performing prophylactic fasciotomy and nerve release, and consideration of gradual instead of acute correction all contribute to preventing complications. Considering and using these diverse skills and resources can improve correction by allowing the surgeon to make surgical decisions based on deformity correction principles rather than compromise the reconstruction because of an outsized fear of unavoidable complications.
